# Sagittal alignment changes and postoperative complications following surgery for adult spinal deformity in patients with Parkinson’s disease: a multi-institutional retrospective cohort study

**DOI:** 10.1186/s12891-021-04233-5

**Published:** 2021-04-16

**Authors:** Atsuyuki Kawabata, Toshitaka Yoshii, Kenichiro Sakai, Takashi Hirai, Masato Yuasa, Hiroyuki Inose, Yu Matsukura, Shingo Morishita, Masaki Tomori, Ichiro Torigoe, Kazuo Kusano, Kazuyuki Otani, Yoshiyasu Arai, Shigeo Shindo, Atsushi Okawa

**Affiliations:** 1 Department of Orthopedic Surgery, Tokyo Medical and Dental University, 1 Chome-5-45 Yushima, Bunkyo City, Tokyo, 113-8510 Japan; 2 Department of Orthopedic Surgery, Saiseikai Kawaguchi General Hospital, 5 Chome-11-5 Nishikawaguchi, Kawaguchi, Saitama, 332-8558 Japan; 3 Department of Orthopedic Surgery, Kudanzaka Hospital, 1 Chome-6-12 Kudanminami, Chiyoda, Tokyo, 102-0074 Japan

**Keywords:** Adult spinal deformity, Parkinson’s disease, Postoperative complications, Sagittal alignment, Sagittal vertical axis, Radiographic parameters

## Abstract

**Background:**

Parkinson’s disease (PD) has been found to increase the risk of postoperative complications in patients with adult spinal deformity (ASD). However, few studies have investigated this by directly comparing patients with PD and those without PD.

**Methods:**

In this multicenter retrospective cohort study, we reviewed all surgically treated ASD patients with at least 2 years of follow-up. Among them, 27 had PD (PD+ group). Clinical data were collected on early and late postoperative complications as well as any revision surgery. Radiographic parameters were evaluated before and immediately after surgery and at final follow-up, including sagittal vertical axis (SVA), thoracic kyphosis, lumbar lordosis, sacral slope, and pelvic tilt. We compared the surgical outcomes and radiographic parameters of PD patients with those of non-PD patients.

**Results:**

For early complications, the PD+ group demonstrated a higher rate of delirium than the PD− group. In terms of late complications, the rate of non-union was significantly higher in the PD+ group. Rates of rod failure and revision surgery due to mechanical complications also tended to be higher, but not significantly, in the PD+ group (*p* = 0.17, *p* = 0.13, respectively). SVA at final follow-up and loss of correction in SVA were significantly higher in the PD+ group.

**Conclusion:**

Extra attention should be paid to perioperative complications, especially delirium, in PD patients undergoing surgery for ASD. Furthermore, loss of correction and rate of non-union were greater in these patients.

## Background

Parkinson’s disease (PD) is a neurodegenerative disorder, and the main symptoms include rigidity, bradykinesia, and gait disorder. As a result of population aging, the number of patients with this age-related disorder is on the rise [1]. In severe cases, PD can lead to postural disorders, such as anterocollis, Pisa syndrome, and camptocormia [2–5]. Studies have reported that these various postural abnormalities can increase susceptibility to development of rigid spinal deformities [2, 3] and that patients with PD develop adult spinal deformity (ASD) more frequently than the general population in the same age group [6].

Recent studies have found that ASD negatively affects health-related quality of life [7, 8]. Surgical treatment has been shown to provide better health-related quality of life outcomes than non-surgical treatment, especially in patients with severe deformity [9]. However, higher complication rates have been reported, with reoperation rates reaching up to 47% [10, 11]. Surgical complications are generally classified as perioperative or late complications. Perioperative complications include epidural hematoma, deep vein thrombosis (DVT), and pulmonary embolism (PE), which usually appear during or soon after surgery. Late complications include junctional kyphosis, rod fractures, and non-union, which usually develop more than 1 month after surgery and are mainly caused by continuous mechanical stress.

Generally, PD patients are presumed to be at higher risk of surgical complications, especially mechanical complications due to postural instability, a higher risk of falls, and decreased bone quality [12–15]. However, to date, only a few small case series have investigated complications after surgery for ASD in PD patients [16–19]. In addition, risk factors for complications and revision surgery in PD patients have not yet been confirmed. Therefore, we conducted this multicenter study in order to investigate 234 surgically treated ASD patients with at least 2 years’ follow-up. We compared surgical outcomes and radiographic parameters of PD patients with those of non-PD patients.

## Methods

This retrospective observational cohort study follows the Strengthening the Reporting of Observational studies in Epidemiology (STROBE) guidelines. We reviewed 234 ASD patients treated surgically from January 2009 to December 2016 at our hospital and affiliated institutions. Institutional review board approval was obtained at each site for the patient enrolment and data collection protocols. Inclusion criteria were age ≥ 21 years at surgery, follow-up period of ≥2 years, and surgery including posterior instrumentation of four spinal levels with sufficient radiographic data. In total, 27 patients with PD were identified. Data were collected on mean PD duration, Hoehn and Yahr (HY) stage, and medication for PD.

A diagnosis of ASD was made if at least one of the following was present: a coronal Cobb angle > 20°, a sagittal vertical axis > 5 cm, pelvic tilt > 25°, and thoracic kyphosis > 60°. Etiologies included degenerative kyphosis/kyphoscoliosis, post-lumbar surgery, and previous vertebral fracture. Operating time and intraoperative blood loss were recorded. Surgical complications are generally classified as early or late. Early complications were defined as those that occurred during or within 1 month following surgery and included neurological disorders, implant failure, DVT, PE, cerebrovascular disease, respiratory disorders, cardiovascular disorders, delirium, surgical site infection, and spinal epidural hematoma. The number of revision surgeries due to perioperative complications was also recorded. Late complications usually occur more than 1 month after surgery and are primarily due to continuous mechanical stress. This stress leads to failure of either the hardware or the vertebra and is defined as a mechanical complication, including, for example, proximal junctional kyphosis (PJK), distal junctional kyphosis, non-union, rod breakage, and vertebral fracture. PJK was defined by two spine surgeons using the following criteria: 1) kyphosis > 10° between the upper-instrumented vertebra and two-level proximal vertebra and 2) a change of ≥10° from the preoperative measurement [20]. Non-union was defined as 1) loss of fixation (e.g., implant breakage/dislodgement of rods or hooks) or a halo (2–4 mm) around a pedicle screw, 2) progression of deformity with or without pain in the fused segments, 3) collapse of the disc space during follow-up or gas in the disc space, and 4) motion (≥3°) on plain flexion/extension radiographs [21]. Mechanical failure was defined as mechanical complications requiring revision surgery.

We collected demographic and clinical data including age, sex, BMI, medical comorbidities, and femoral neck bone mineral density (BMD). Measurements on radiographs included SVA, thoracic kyphosis (T4–T10), thoracolumbar kyphosis (T10–L2), lumbar lordosis (LL; L1–S1), sacral slope, pelvic incidence (PI), and pelvic tilt. These parameters were evaluated in the standing position both before and at 4 weeks post-surgery. X-ray images were also evaluated at the final follow-up. A three-column osteotomy was defined as a procedure using pedicle subtraction osteotomy or vertebral column resection. A radiographic parameter of − 10° < PI−LL < 10° was defined as ideal alignment based on the SRS-Schwab ASD classification.

We compared the surgical outcomes and radiographic parameters of PD patients (the PD+ group) with those of the non-PD patients (the PD− group). Statistical analysis was performed using IBM SPSS Statistics for Macintosh, Version 25.0 (IBM Corp, Armonk, NY). We used a paired t-test or chi-squared test to compare the PD+ and the PD− groups. Also, t-tests were used to compare the means of continuous variables, and chi-square tests were used to compare the proportions of categorical variables between the groups. A *p*-value < 0.05 was considered statistically significant. Missing values were imputed using the last observation carried forward method. We also matched the background data using propensity score matching, a technique that has been extensively used to adjust for known confounding biases [22–24]. The propensity score for mechanical failure was initially calculated using the following variables: patient age and sex, BMI, fusion levels, and preoperative SVA. We performed the procedure using a logistic regression model. The C-statistic suggested that the fit was 0.67, which is a fairly good score. Patients in the two study groups were matched based on propensity scores, with the condition that the caliper be lower than 0.2. Twenty-four pairs of patients with and without PD were created after matching. Postoperative complications in the matched cases were compared between the two groups.

## Results

Table 1 shows the patient characteristics of the PD+ group and the PD− group. There was no significant difference between the two groups with respect to age, sex, BMI, or BMD. The number of fixed spinal levels was significantly higher in the PD+ group than in the PD− group (9.3 ± 2.6 vs 7.8 ± 1.9, *p* = 0.006), and the rates of three-column osteotomy tended to be higher in the PD+ group (63.0% vs 48.5%, *p* = 0.14). For preoperative radiographic parameters, SVA was significantly higher in the PD+ group than in the PD− group (196.0 ± 63.8 mm vs 135.4 ± 69.9 mm, *p* < 0.001). Other parameters were not significantly different between the groups. In terms of etiology, ASD caused by previous vertebral fracture was significantly higher in the PD+ group than in the PD− group (previous vertebral fracture/degenerative/post lumbar surgery: 38.5%/53.8%/7.7% vs 10.1%/64.7%/25.1%, *p* = 0.005). Of the 27 patients in the PD+ group, 23 were taking dopamine precursors, 8 were taking dopamine agonists, and 4 were not taking any medication for PD.

**Table 1:** Demographic and clinical characteristics of the two study groups

	PD+ group	PD− group	***p***-value
Cases, n	27	206	–
Age at surgery (years)	70.6 ± 6.3	72.5 ± 8.5	0.18
Sex (male/female, n)	5/22	32/174	0.58
BMI	22.8 ± 4.2	23.2 ± 3.7	0.59
BMD (T-score)	−1.900 ± 1.249	− 1.863 ± 0.924	0.93
Medications for PD			
Dopamine precursors (yes/no)	23/4 (85.2%)	–	–
Dopamine agonists (yes/no)	8/19 (29.6%)	–	–
Monoamine oxidase type B inhibitors (yes/no)	6/21 (22.2%)	–	–
Other	4/23 (14.8%)	–	–
Medical comorbidities			
Diabetes (yes/no)	1/26 (3.7%)	22/184 (10.7%)	0.49
Rheumatoid arthritis (yes/no)	0/27	9/197 (4.4%)	0.60
Renal dysfunction (yes/no)	0/27	19/187 (9.2%)	0.14
Cardiovascular disease (yes/no)	2/25 (7.4%)	26/180 (12.6%)	0.75
Cerebrovascular disease (yes/no)	5/22 (18.5%)	14/192 (6.8%)	0.053
Respiratory disease (yes/no)	0/27	21/185 (10.2%)	0.14
Follow-up duration (months)	50.3 ± 16.2	49.0 ± 14.1	0.68
Number of fixed levels	9.3 ± 2.6	7.8 ± 1.9	0.006
3CO (yes/no)	17/10	100/106	0.14
Etiology (post fracture/degenerative/previous lumbar fusion)	10/15/2	21/133/52	0.001
Fix to sacrum (yes/no)	22/5 (81.5%)	174/32 (84.1%)	0.78
Pre SVA (mm)	196.0 ± 63.7	135.4 ± 69.9	< 0.001
Pre LL (°)	2.1 ± 19.3	3.5 ± 20.5	0.75
Pre TLK (°)	18.1 ± 16.0	19.2 ± 17.0	0.28
Pre TK (°)	24.1 ± 15.4	27.4 ± 20.0	0.37
Pre SS (°)	17.1 ± 8.0	14.7 ± 11.4	0.34
Pre PT (°)	36.6 ± 8.6	34.5 ± 13.2	0.47
PI (°)	53.6 ± 9.6	49.8 ± 9.1	0.088

*3CO* Three-column osteotomy, *BMD* Bone mineral density, *BMI* Bone mass index, *LL* Lumbar lordosis, *PD* Parkinson’s disease, *PI* Pelvic incidence, *PT* Pelvic tilt, *SS* Sacral slope, *SVA* Sagittal vertical axis, *TK* Thoracic kyphosis.

Table 2 shows the surgical invasiveness and postoperative complications for both groups. There was no significant difference in operating time or intraoperative blood loss between the groups. For early complications, the PD+ group showed a higher rate of delirium than the PD− group. In the PD+ group, rates of DVT and PE tended to be higher (DVT: 14.8% vs 6.8%, *p* = 0.12; PE: 3.7% vs 0.5%, *p* = 0.081); the difference was not statistically significant. There were no differences between the groups in the rates of other complications, including neurological deficits, implant failure, cerebrovascular disorders, respiratory disorders, cardiovascular disorders, and surgical site infections. There was no significant difference in the rate of revision surgery due to early complications. For late complications, the non-union rate was significantly higher in the PD+ group (37.0% vs 19.4%; *p* = 0.017). Rates of rod failure and revision surgery due to mechanical complications also tended to be higher, albeit not significantly, in the PD+ group (rod failure: 25.9% vs 13.1%, *p* = 0.174; revision surgery: 33.3% vs 18.0%, *p* = 0.13).

**Table 2:** Postoperative complications in the two study groups

	PD+ group	PD− group	***p***-value
Cases, n	27	206	–
Operating time (min)	490 ± 154	454 ± 113	0.14
Blood loss (g)	2069 ± 1336	1984 ± 1422	0.28
Early complications			
Neurological (yes/no)	3/24 (11.1%)	23/183 (11.2%)	0.96
Implant failure (yes/no)	2/25 (7.4%)	20/186 (9.7%)	0.74
DVT (yes/no)	4/23 (14.8%)	14/192 (6.8%)	0.12
PE (yes/no)	1/26 (3.7%)	1/205 (0.5%)	0.081
Cerebrovascular disorder (yes/no)	0/27 (0%)	2/204 (1.0%)	0.61
Respiratory disorder (yes/no)	1/26 (3.7%)	7/199 (3.4%)	0.91
Cardiovascular disorder (yes/no)	0/27 (0%)	6/200 (2.9%)	0.38
Delirium (yes/no)	7/20 (25.9%)	15/191 (7.3%)	0.001
Surgical site infection (yes/no)	1/26 (3.7%)	6/200 (2.9%)	0.79
Spinal epidural hematoma (yes/no)	0/27 (0%)	14/192 (6.8%)	0.17
Late complications			
Mechanical complication (yes/no)	15/12 (55.6%)	99/107 (48.1%)	0.59
Rod failure (yes/no)	7/20 (25.9%)	27/179 (13.1%)	0.17
Non-union	10/17 (37.0%)	34/172 (16.5%)	0.017
PJK (yes/no)	8/19 (29.6%)	55/151 (26.7%)	0.82
DJK (yes/no)	3/24 (11.1%)	15/191 (7.3%)	0.95
Vertebral fracture (yes/no)	7/20 (25.9%)	43/163 (20.9%)	0.81
Revision (yes/no)	9/18 (33.3%)	37/169 (18.0%)	0.13

*DJK* Distal junction kyphosis, *DVT* Deep vein thrombosis, *PD* Parkinson’s disease, *PE* Pulmonary embolism, *PJK* Proximal junction kyphosis.

Table 3 summarizes the changes in the radiographic parameters after surgery and at the final follow-up. Postoperative SVA was similar between the two groups, although the preoperative SVA was much higher in the PD+ group. The change in SVA on radiographs obtained before and after surgery was significantly greater in the PD+ group (− 142.5 ± 82.0 mm vs − 94.6 ± 69.7 mm, *p* = 0.014). Furthermore, SVA at the final follow-up tended to be greater in the PD+ group (*p* = 0.062). Loss of correction of SVA also tended to be more common in the PD+ group (*p* = 0.11). No significant difference were found for any other radiographic parameter, including the proposed ideal alignment target of PI–LL < 10.

**Table 3:** Postoperative radiographic parameters in the two study groups

	PD+	PD−	***P***-value
Cases, n	27	207	–
Post SVA (mm)	53.5 ± 41.6	40.0 ± 46.2	0.17
Change in SVA (post-pre) (mm)	− 142.5 ± 82.0	−94.6 ± 69.7	0.003
Final SVA (mm)	89.2 ± 68.1	59.9 ± 52.2	0.062
Loss of SVA correction (mm)	35.7 ± 62.1	19.2 ± 43.4	0.11
Post LL (°)	41.4 ± 10.8	39.7 ± 11.3	0.64
Final LL (°)	38.6 ± 12.3	36.4 ± 12.5	0.74
LL correction loss (°)	−0.64 ± 20.9	−0.20 ± 21.3	0.93
Post TK **(**°**)**	37.1 ± 12.9	36.5 ± 14.5	0.85
Final TK (°)	44.3 ± 14.8	43.0 ± 16.5	0.70
TK correction loss (°)	7.3 ± 7.1	6.5 ± 10.6	0.87
Post SS (°)	27.1 ± 6.6	26.2 ± 8.6	0.56
Final SS (°)	27.4 ± 9.6	24.7 ± 17.3	0.48
SS correction loss (°)	−0.3 ± 6.4	1.7 ± 16.8	0.58
Post PT (°)	26.5 ± 9.4	23.0 ± 10.2	0.12
Final PT (°)	26.2 ± 11.3	24.7 ± 19.0	0.59
−10 < post PI-LL < 10 (yes/no)	12/15	108/99	0.83

*LL* Lumbar lordosis, *PD* Parkinson’s disease, *PI* Pelvic incidence, *PT* Pelvic tilt, *SS* Sacral slope, *SVA* Sagittal vertical axis, *TK* Thoracic kyphosis.

Table 4 presents the preoperative demographics, postoperative radiographic parameters, and surgical characteristics of the PD+ group according to whether revision surgery was required. No significant difference was found in any radiographic parameter between the two subgroups. However, the duration of PD was significantly longer in the revision subgroup than in the non-revision subgroup (87.0 ± 56.9 months vs 32.5 ± 48.0 months, *p* = 0.037). HY stage was higher in the revision subgroup than in the non-revision subgroup but was not significantly different (2.8 ± 1.2 vs 2.2 ± 1.3, *p* = 0.29).

**Table 4:** Postoperative radiographic parameters and demographics in the PD+ group according to whether revision surgery was required

	Revision	No revision	***p***-value
Cases, n	9	18	–
Pre SVA (mm)	187.0 ± 62.3	193.4 ± 63.6	0.84
Post SVA (mm)	60.2 ± 27.4	57.7 ± 39.2	0.87
Pre LL (°)	−3.5 ± 27.3	4.9 ± 16.3	0.51
Post LL (°)	33.7 ± 13.4	44.6 ± 8.6	0.11
Pre TLK (°)	9.3 ± 6.9	11.2 ± 12.5	0.26
Post TLK (°)	11.4 ± 11.4	0.2 ± 11.6	0.074
Pre TK (°)	15.0 ± 16.0	26.3 ± 13.7	0.17
Post TK (°)	30.0 ± 18.5	40.1 ± 9.8	0.25
Pre SS (°)	16.2 ± 11.8	17.0 ± 6.5	0.87
Post SS (°)	24.3 ± 7.8	28.5 ± 6.2	0.28
Pre PT (°)	35.5 ± 7.3	37.0 ± 9.3	0.71
Post PT (°)	27.3 ± 5.2	26.2 ± 10.7	0.74
PI (°)	51.7 ± 8.7	54.6 ± 10.4	0.53
Age at surgery (years)	68.5 ± 3.7	71.6 ± 6.5	0.19
BMI	21.9 ± 3.7	23.8 ± 4.3	0.33
Number of fixed levels	9.0 ± 2.5	10.2 ± 2.5	0.35
Duration of PD (months)	87.0 ± 56.9	32.5 ± 48.0	0.037
Hoehn and Yahr stage	2.8 ± 1.2	2.2 ± 1.3	0.29
BMD (T-score)	−2.1 ± 1.7	− 1.8 ± 1.2	0.81

*BMD* Bone mineral density, *BMI* Bone mass index, *LL* Lumbar lordosis, *PD* Parkinson’s disease, *PI* Pelvic incidence, *PT* Pelvic tilt, *SS* Sacral slope, *SVA* Sagittal vertical axis, *TLK* Thoracolumbar kyphosis, *TK* Thoracic kyphosis.

Table 5 shows the demographics and postoperative complications in the PD+ and PD− groups after propensity score matching. Twenty-four pairs of patients, one with PD and one without PD, were created after matching. Age at surgery, sex, BMI, BMD, fusion levels, preoperative SVA, and LL were almost completely matched. Revision surgery due to mechanical complications tended to be higher, but not significantly, in the PD+ group (33.3% vs 12.5%, *p* = 0.086).

**Table 5:** Demographic and postoperative complications in the study groups after propensity score matching

	PD+ group	PD− group	***p***-value
Cases, n	24	24	–
Age at surgery (years)	70.8 ± 6.5	70.6 ± 11.5	0.95
Sex (male/female), m	5/19	3/21	0.70
BMI	23.2 ± 4.1	24.0 ± 4.1	0.49
BMD (T-score)	−1.7 ± 1.2	− 2.0 ± 0.75	0.55
Number of fixed levels	9.0 ± 2.4	9.0 ± 2.0	0.95
3CO (yes/no)	7/17	11/13	0.37
Fix to sacrum (yes/no)	24/0	24/0	–
Pre SVA (mm)	185.7 ± 62.4	182.9 ± 76.9	0.89
Pre LL (°)	0.04 ± 21.8	−1.0 ± 20.0	0.86
Mechanical complication (yes/no)	14/10 (58.3%)	11/13 (45.8%)	0.38
Rod failure (yes/no)	6/18 (25.0%)	4/20 (16.7%)	0.48
Non-union	10/14 (41.7%)	4/20 (16.7%)	0.059
PJK (yes/no)	7/17 (29.2%)	9/15 (37.5%)	0.54
DJK (yes/no)	2/22 (8.3%)	0/24 (0%)	0.15
Vertebral fracture (yes/no)	6/18 (25.0%)	8/16 (33.3%)	0.53
Revision (yes/no)	8/16 (33.3%)	3/21 (12.5%)	0.086
Post SVA (mm)	50.0 ± 46.5	48.3 ± 42.8	0.89
Post LL (°)	41.7 ± 10.2	41.9 ± 12.1	0.95
Final SVA (mm)	89.9 ± 62.2	65.6 ± 46.9	0.13
Final LL (°)	39.4 ± 12.1	38.3 ± 14.2	0.95
SVA correction loss (mm)	39.8 ± 48.7	17.3 ± 38.8	0.08
LL correction loss (°)	−3.3 ± 20.8	−3.5 ± 19.9	0.97

*3CO* Three-column osteotomy, *BMD* Bone mineral density, *BMI* Bone mass index, *DJK* Distal junction kyphosis, *LL* Lumbar lordosis, *PD* Parkinson’s disease, *PJK* Proximal junction kyphosis, *SVA* Sagittal vertical axis.

## Discussion

Patients with PD have been reported to have more postoperative complications due to various musculoskeletal problems. High rates of perioperative and postoperative complications following hip and knee surgery in patients with PD have been found in studies using a nationwide inpatient database [25, 26]. Furthermore, surgical studies on the PD population have suggested higher rates of postoperative medical complications, including pneumonia, delirium, and sepsis [5]. However, only a few case series have examined the relationship between PD and complications following corrective surgery for ASD. Furthermore, there have been few detailed comparisons of radiographic parameters between patients with and without PD. Therefore, in this multicenter study, we retrospectively compared both postoperative complications and radiographic parameters in patients with ASD according to whether they had PD. This study has three strengths: 1) detailed data on radiographic parameters and early/late postoperative complications were collected; 2) patients with and without PD were compared using both crude analysis and propensity matching analysis; 3) all patients were followed up for at least 2 years; and, 4) the number of samples included had adequate statistical power.

In this study, the rate of delirium was significantly higher and the rates of PE and DVT tended to be higher in patients with PD. Our finding of a higher rate of delirium in patients with PD is consistent with that of Watanabe et al., who reported that postoperative delirium was more common in patients with PD (23.1%) compared with a control group (3.4%) [27]. Our patients who developed delirium were taking at least one medication for PD and had an HY stage of more than 2, which suggests that this higher rate of delirium was possibly due to use of PD drugs as well as the neurodegenerative state in patients with PD. Although delirium is thought to be a reversible condition, there have been reports of postoperative delirium leading to prolonged hospitalization and increased morbidity and mortality rates. Further complications may also occur due to falls or movement beyond the limits of restriction. Therefore, patients with PD should be investigated carefully for modifiable risk factors, including management of sedation, deliriogenic medications, immobility, and sleep disruption. The incidence of thrombotic events was relatively high in our patients with PD but was not significantly different from that in patients without PD. However, a previous study in Japan that included data from a large nationwide database found a significantly increased risk of PE in patients with PD [28]. PE is recognized as a possible adverse reaction to dopamine precursors, such as levodopa [29]. Moreover, Yamane et al. reported a higher incidence (20%) of DVT in patients with PD who had a postural abnormality [30]. Given that patients who undergo corrective surgery for ASD are generally at higher risk of PE due to the long operating time and the lengthy immobilization period after surgery, the risk of PE in patients undergoing surgery for ASD is likely to be high. Therefore, surgeons should consider a thrombotic event when a patient develops chest pain and dyspnea after surgery, particularly if they have PD. Preoperative screening of the D-dimer level or an ultrasound examination for DVT could be a viable preventative strategy.

In this study, we found a significantly higher preoperative SVA and greater correction of SVA in our PD+ group. We also found that the SVA at final follow-up was higher in the PD+ group. The loss of correction of SVA was also slightly higher in the PD+ group, even though more vertebral segments were fused in this group. This finding suggests that the deformities in the PD+ group were severe but essentially flexible and were largely corrected by the surgery. However, the improved SVA was not well maintained in this group. The exact reasons for the increased SVA after surgery in patients with PD are unknown. However, the stooping posture associated with PD could in itself cause deterioration in overall sagittal balance, and this may be one of the causes of the poorer outcome in patients with PD. Kawaguchi et al. reported that longer fusion (up to the T4 level) achieved a good clinical outcome in a patient with PD after corrective surgery from L1 to S1 had been unsuccessful [31]. Watanabe et al. reported that patients with PD who were surgically treated for ASD had poor clinical outcomes and high rates of non-union and adjacent segment disease [32]. Therefore, it is important to consider possible prevention strategies, including fusion of a greater number of segments.

In this study, the revision rate due to mechanical complications in the PD+ group was 33.3% and almost double that in the PD− group. This finding is similar to that in a study by Sheu et al., who reported that 29% of 66 patients with PD who underwent instrumented thoracolumbar or lumbar surgery for degeneration or deformity required revision surgery due to mechanical complications [15]. Bouyer et al. reported a high revision rate of 42% in 48 patients with PD who underwent surgery for ASD; the revision surgery was performed for mechanical complications in 89% of cases [18]. PJK has been reported to be significantly more common in patients with PD [5]. However, a diagnosis of PD had no significant impact on the PJK rate in this study, although rates of non-union and rod failure were higher in our PD+ group. Even after matching for fusion levels and preoperative SVA using propensity scores, the rate of non-union tended to be higher in the PD+ group. Non-union can initiate pain and hardware issues, such as loosening of screws and rod fracture. Therefore, the surgeon should consider prevention strategies in patients with PD, such as administration of teriparatide.

Several studies have investigated the risk factors for revision surgery in patients with PD. Schroeder et al. reported that an HY stage > 2, diabetes mellitus, treatment for osteoporosis, and a combined anterior and posterior surgical approach were risk factors for revision surgery in 94 lumbar spine surgeries [14]. According to Sheu et al., HY stage > 2, a history of cancer, osteoporosis, and three-column osteotomy were risk factors for revision surgery [15]. Evaluation of walking ability using the HY scale can be affected by symptoms of ASD, so the stage itself may not reflect the exact severity of PD in patients undergoing ASD. However, in our study, the revision subgroup tended to have a higher HY stage and a longer duration of PD. Therefore, it is important to consider both duration and severity of PD when performing surgery in these patients.

This study has several limitations. First, the surgeons were able to select the surgical procedure based on PD status, which would introduce selection bias. Second, because the study had a multicenter design, we used simple criteria to identify mechanical complications, namely, plain radiographs. Hence, the nonunion rate may have been underestimated. Third, the patients in the PD+ and PD− groups had different background characteristics. This problem was dealt with by propensity score matching to allow for comparison of the two groups. Fourth, the study was retrospective in nature, and the number of patients with PD was relatively low. When considering the non-union rates in our study groups, the effect size (w) was 0.376. A subsequent post hoc analysis showed that the statistical power for the chi-square test was β = 0.99 when the type I error rate (α) was set at 0.05 (G*power 3.1). Therefore, we consider that the main results of this study, including the significant between-group difference in the non-union rate, were based on an adequate sample size. However, this does not necessarily mean that the sample size was large enough for all of the analyses. Better designed studies that include a larger sample size are needed in the future.

Despite the above-mentioned limitations, this study yielded the following important findings: 1) patients with PD were at greater risk of developing delirium in the early postoperative period; 2) the non-union rate was significantly higher in patients with PD; and 3) the rate of revision surgery tended to be higher in patients with PD in the late postoperative period.

## Conclusion

Postoperative delirium was common in patients with PD. Late complications included rod fracture, non-union, and revision surgery, and occurred more frequently in patients with PD. The rate of revision surgery due to mechanical complications in patients with PD was approximately double that of their counterparts without PD. In patients with PD, the SVA was significantly larger before surgery and at final follow-up, as was the loss of correction of SVA, suggesting that PD-specific postural abnormalities are involved in deterioration of the sagittal parameters. The duration of PD was significantly longer in patients who underwent revision surgery than in those who did not.

## Data Availability

The datasets generated during and/or analyzed during the current study are available from the corresponding author on reasonable request.

## Abbreviations

ASD: Adult spinal deformity
BMI: Body mass index;
BMD: Bone mineral density
DVT: Deep vein thrombosis
DJK: Distal junctional kyphosis
HY: Hoehn and Yahr
LL: L1–S1 lumbar lordosis
PD: Parkinson’s disease
PI: Pelvic incidence
PJK: Proximal junctional kyphosis
PE: Pulmonary embolism
T4–T10: Thoracic kyphosis
UIV: Upper instrumented vertebra
